# Fatty Acids Profile and Healthy Lipids Indices of Native Mexican Guajolote Meat Treated to Two Heat Treatments

**DOI:** 10.3390/foods11101509

**Published:** 2022-05-22

**Authors:** Francisco A. Cigarroa-Vázquez, Lorenzo Danilo Granados-Rivera, Rodrigo Portillo-Salgado, Joel Ventura-Ríos, William Esponda-Hernández, José A. Hernández-Marín, Alvar A. Cruz-Tamayo, Yuridia Bautista-Martinez

**Affiliations:** 1 School of Agricultural Studies Mezcalapa, Autonomous University of Chiapas, Copainalá 29620, Mexico; antonio.cigarroa@unach.mx (F.A.C.-V.); william.esponda@unach.mx (W.E.-H.); 2 Experimental Field—General Teran, National Institute of Forestry, Agricultural and Livestock Research, General Teran 67400, Mexico; 3 Livestock Program, College of Postgraduates, Texcoco 56264, Mexico; rps_1303@hotmail.com; 4 School of Veterinary Medicine and Animal Husbandry, Autonomous Agrarian University Antonio Narro, Saltillo 25315, Mexico; joelventur@gmail.com; 5 Department of Veterinary and Zootechnics, University of Guanajuato, Irapuato 38096, Mexico; jahmarin@ugto.mx; 6 Faculty of Agricultural Sciences, Autonomous University of Campeche, Escarcega 24350, Mexico; alvar_cruz@hotmail.com; 7 School of Veterinary Medicine and Animal Husbandry, Autonomous University of Tamaulipas, Ciudad Victoria 87000, Mexico

**Keywords:** native guajolote, heat treatment, fatty acids, health indices

## Abstract

**Simple Summary:**

The native guajolote meat is an important dietary source of proteins of high biological value for the rural population of Mexico. However, many of its quality characteristics are still unknown. Therefore, the fatty acids (FAs) profile and nutritional indices of breast and leg meat of native guajolote subjected to two heat treatments (boiled and baked) were evaluated. The heat treatments increased the concentration of saturated (SFA) and monounsaturated FA (MUFA) in the meat; in contrast, the concentration of polyunsaturated FA (PUFA) decreased. Likewise, the dietary FA index and the atherogenic index increased in guajolote meat from the effect of the heat treatments, while the essential and hypercholesterolemic FA indices decreased. Based on the results obtained, heat treatments increase the content of SFA and MUFA in breast and leg meat of native guajolote. Baking is less favorable for both types of muscle.

**Abstract:**

Meat is a complex food with a structured nutritional composition that makes it an essential component of the human diet. In particular, the meat of native guajolote that is traditionally raised in natural conditions is an important dietary source of proteins of high biological value for the rural population of Mexico. The study aimed to evaluate fatty acids (FAs) profile and nutritional indices of breast and leg meat of native guajolote subjected to two heat treatments. For the study, a total of sixty muscle samples (30 breast meat and 30 leg meat) from adult male native guajolotes were used. The FA profile and nutritional indices were evaluated in raw meat (control) and meat subjected to two heat treatments (boiled and baked). The heat treatments, independently of the type of muscle, increased (*p* ≤ 0.05) the concentration of saturated (SFA) and monounsaturated FA (MUFA); in contrast, polyunsaturated FA (PUFA) decreased. Likewise, the dietary FA index, which has a negative hypercholesterolemic effect, and the atherogenic index increased in guajolote meat from the effect of the heat treatments, while the essential and undesirable hypercholesterolemic FA indices decreased. In conclusion, heat treatments increase the content of SFA and MUFAs in breast and leg meat of native guajolote. Boiling or baking the meat deteriorates PUFAs but increases the nutritional indices. The present investigation would provide valuable information for the guajolote meat product processing.

## 1. Introduction

In the world, the production and consumption of poultry meat are growing steadily, with chicken meat as the preferred source of animal protein among consumers [1]. Chicken meat is obtained through the intensive production of broilers of fast-growing breeds [2]. However, in Mexico, the rural population has low access to this type of meat, and its main source of animal protein is meat from poultry raised traditionally in the open air, such as Creole hens and chickens, ducks, geese, and native guajolotes [3].

In particular, raising the native guajolote (*Meleagris gallopavo gallopavo*) provides rural farmers with high-quality protein given its carcass yield, which is around 79%, much higher than other farm animals [4]. In this regard, Cigarroa et al. [5] determined that the conditions of free-range breeding traditions of the native guajolote generate an environment of wellbeing for the birds, which in turn guarantees the obtaining of products of better nutritional quality. 

On the other hand, the guajolote constitutes a genetic reservoir with unique characteristics of economic importance and adaptability [6]. This is due to the fact that it has greater genetic variability compared to the breeds commercial turkeys [7,8]. However, despite the important socio-cultural and genetic value of the Guajolote, until now, it has not been reported or officially registered as a native breed of Mexico, and even less has it been included in selection and genetic improvement programs. This is due to the fact that many of its characteristics, including its nutritional quality of meat, have not been described.

In this regard, it is well-known that consumer acceptance of meat is strongly influenced by the cooking method or heat treatment used for meat processing, and immersion cooking and baking are two of the most commonly used thermal methods in food preparation. Therefore, the changes in the physicochemical properties of meat due to the cooking effect should be studied [9].

In this sense, and considering the increase in poultry production in an alternative system that is also friendly to the environment, mindful of animal welfare, and that contributes to the food sustainability of rural communities [10] through the production of quality foods, such as lipids and antioxidants [11], it is necessary to promote the alternative rearing of the native guajolote, for which it is necessary to know its production method and the characteristics of the products in order to improve this type of alternative breeding that helps the sustainable development of rural communities and in turn help conserve the native Mexican guajolote, an important genetic reservoir for itself and for commercial turkey breeds around the world.

Therefore, the aim of this study was to evaluate fatty acids (FAs) profile and nutritional indices of breast and leg meat of native guajolote (*Meleagris g. gallopavo*) subjected to two heat treatments (boiled and baked) in order to generate unpublished information on the quality of meat from this type of animal and its possible implications for the health of consumers.

## 2. Materials and Methods

### 2.1. Study Area

The study was carried out in the Animal Nutrition Laboratory of the Livestock Program of the Colegio de Postgraduados, Campus Montecillo, located in Texcoco, the State of Mexico, at 19°29′ NL and 98°53′ WL, at an altitude of 2240 masl.

### 2.2. Meat Samples

The experimental material was the breast (*Pectoralis major*) and leg muscles from 10 adult, male 32-week-old guajolotes, with an average body weight of 5.4 ± 1.4 kg. The birds were raised under extensive traditional conditions in production units of rural communities in the municipality of Villaflores, Chiapas, Mexico [12]. To obtain meat samples, all the birds were sacrificed on the same day after a 10 h fasting period, during which they received only clean water. The slaughter was carried out in accordance with the Official Mexican Standards (NOM-008-ZOO-1994, NOM-009-ZOO-1994 and NOM-033-SAG/ZOO-2014) established for the humane slaughter of animals intended for meat production. The birds were humanely killed by exsanguination, and the carcasses were then scalded in hot water (60–65 °C) for 2 min for manual plucking. The eviscerated carcasses were placed in a refrigerator at 4 °C for 24 h in a modified atmosphere. Subsequently, the breast and leg muscles (without skin) were cut and standardized in terms of thickness and weight (10 g per sample). Twenty guajolote meat samples were used (10 breast and 10 leg muscles) for analysis as raw meat and subjected to two heat treatments for a total of 60 samples.

### 2.3. Thermal Treatments

An analysis was performed on raw meat with the aim to identify the loss of fatty acids with the heat treatments, boiling, and baking, similar to what is commonly done when guajolote meat is prepared in rural communities where it is consumed. No food additives were added to any heat treatment. Each meat sample had a weight of 100 g.

### 2.4. Immersion Cooking

Each breast and leg muscle was tagged with an edible food-tying thread and submerged in a container with water at a temperature of 90 °C and cooked for 30 min.

### 2.5. Oven

The breast and leg muscles were placed in aluminum trays with wells for their identification, and later, they were baked in a conventional oven, which was preheated to 180 °C, after which the samples were introduced and kept at a constant temperature of 200 °C for 25 min.

### 2.6. Fatty Acid Composition

The extraction and methylation of fatty acids from breast and leg muscles in raw, boiled, and baked meat were determined using the technique proposed by Palmquist and Jenkins [13], in which fatty acids are presented in the form of methyl esters. Fatty acid methyl esters were determined through gas chromatography (Hewlett Packard 6890) equipped with an automatic injector and a silica capillary column (100 m × 0.25 mm × 0.20 μm thickness, Sp-2560, Supelco, Pennsylvania, USA). The identification of the fatty acids was done by comparing the retention times of each peak obtained from the chromatogram, with a standard of 37 methyl ester components (37 Component FAME Mix, Catalog No. -U, Supelco). The results were expressed as individual percentages of the fatty acid with respect to the total concentration.

### 2.7. Calculation of Lipid Indices for Health

To calculate nutritional indices of lipids, the fatty acid profile of guajolote breast and leg muscles was used through the following equations:

Nutritive value index (NVI) = (C18:0 + C18:1)/C16:0 [14];
Atherogenic index (AI) = (C12:0 + 4 × C14:0 + C16:0)/∑UFA [15];
Thrombogenic index (TI) = (C14:0 + C16:0 + C18:0)/[(0.5 × ∑MUFA) + (0.5 × ∑PUFAn − 6) + (3 × ∑PUFAn − 3) + (∑PUFAn − 3/∑n − 6)] [15];
Dietary fatty acids that have an undesirable hypercholesterolemic effect in humans (OFA) = (C14:0 + C16:0) [15]; Dietary fatty acids that have a desirable neutral hypocholesterolemic effect in humans (DFA) = (∑MUFA + ∑PUFA + C18:0) [16].

Similarly, total unsaturated (UFA), saturated (SFA), monounsaturated (MUFA), essential (EFA:C18:2 + C18:3 + C20:4), polyunsaturated (PUFA) fatty acids, and the following ratios were determined: ∑DFA/∑OFA, ∑UFA/∑SFA, and ∑PUFA/∑SFA.

### 2.8. Statistical Analysis

The normal distribution of the data and the homogeneity of variances of the results obtained were verified prior to the statistical analysis by means of the Kolmogorov–Smirnoff test and the Levenne test, respectively. When homoscedasticity or normality were not achieved, the inverse or logarithmic arcsine square root transformation was applied to reach or approximate a normal distribution, and later, an analysis of variance was used through a complete design with randomized treatments with factorial arrangement, using each combination of muscle (breast and leg) and heat treatment (raw, boiled, and baked). The treatments (T) were as follows: T1 (*n* = 10): raw breast (BRM); T2 (*n* = 10): raw leg (LRM); T3 (*n* = 10): boiled breast (BBL); T4 (*n* = 10): boiled leg (LBL); T5 (*n* = 10): baked breast (BBK); and T6 (*n* = 10): baked leg (LBK). The effects of muscle type, heat treatment, and their interaction were identified. The statistical significance of the differences between the means of the treatments was verified using the Tukey test at the level of significance *p* ≤ 0.05, with the use of the SAS statistical software.

## 3. Results

Table 1 shows the content of saturated fatty acids in the breast and legs muscles of native guajolote subjected to two heat treatments (boiled and baked).

Differences in SFA by effect of heat treatment (*p* < 0.001) and muscle types (*p* < 0.05) were observed in the fatty acids C:12:0, C:16:0, and C18:0 without changes in the interactions between muscles and heat treatment (*p* > 0.05). With respect to the sum of SFA, it was higher (*p* < 0.05) for both muscles with the baked heat treatment (BBK: 42.2% and LBK: 42.35%), followed by the boiled heat treatment (BBL: 41.33% and LBL: 40.52%) and, finally, lower concentrations in raw muscles (BRM: 36.74% and LRM: 37.5%).

The baked cooking treatment (BBK and LBK) caused the C:12:0 (lauric acid) to increase by 56% in relation to the value of raw meat (BRM and LRM) and by 44% with respect to the value of boiled meat (BBL and LBL). The lowest content values of this fatty acid were for leg muscle (LRM, LBL, and LBK) and the highest for breast muscle (BRM, BBL, and BBK). Low proportions of C:14:0 (myristic acid) were found, with minimum values of 0.59% and maximum 0.64%, without differences (*p* > 0.05) in all treatments. The SFA with the highest content in the breast and leg muscles was C:16:0 (palmitic acid), which increased in both heat treatments (boiled and baked) compared to raw meat (3% and 10%, respectively). The C:18:0 (stearic acid) maintained a similar content in the thermal treatments, namely 13.1% in BBL, 13.05% BBK, 13.5% in LBL, and 13.87% in LBK, but lower than these were BRM and LRM, with 9.49% and 12.39%, respectively. Regarding fatty acids C:15:0, C17:0, and C20:0, no differences were observed (*p* > 0.05) in relation to heat treatment and raw meat as well as for both muscles evaluated. The baking heat treatments, namely LBK (42.35%) and BBK (42.2%), were higher than the boiled heat treatments, namely BBL (41.33%) and LBL (40.52%), and lastly the raw muscles, namely LRM (37.5%) and BRM (36.74%), with regard to the ∑SFA.

The results obtained for the MUFAs for each treatment are shown in Table 2.

Statistical differences (*p* < 0.001) were found from the effects of heat treatments on C:14:1, C:17:1, C18:1 Cis, C24:1, as well as differences (*p* < 0.001) from the effects of muscle type on C16:1 and the ∑MUFA. No differences (*p* > 0.05) were found between muscle types and heat treatment. Myristoleic acid (C:14:1) showed a higher concentration in both muscles that received baked treatment (BBK and LBK: 0.15%); however, it did not undergo changes in relation to BRM (0.12%) with BBL (0.12%), but it did in the boiled treatment (LBL, 0.10%) and for the raw muscle (LRM, 0.09%).

Statistical differences (*p* <0.05) in palmitoleic acid (C16:1) were only found from the effects of muscle type, with a maximum value of 0.42% for BBK. CisHeptadecanoic acid (C17:1) in BBK was similar to LBK (*p* > 0.05) but significantly different (*p* ≤ 0.05) from the rest of the treatments (BRM, LRM BBL, LBL). Oleic acid (C:18:1) had a slight decrease in the baked treatments, BBK (25.9%) and LBK (22.43%) compared to raw muscles (BRM: 26.51% and LRM: 23.86%, *p* ≤ 0.05). The highest content of eladic acid (C:18:1 trans) was found in raw leg (LRM:0.29%), and the lowest content was for the boiled treatments (BBL and LBL: 0.17%), which indicated a loss of this fatty acid to the application of the treatment. There were no differences (*p* > 0.05) in eicosenoic acid (C:20:1); all treatments had similar behavior. Nervonic acid (C:24:1) presented higher values in the raw treatments, namely 0.64% and 0.42% for BRM and LRM, respectively, followed by the treatments for BBL at 0.43% and LBL at 0.41%, with lower content for the BBK (0.36%) and LBK (0.37%) treatments evidencing a decrease in these fatty acids when applying a heat treatment whether boiled or baked. Finally, differences (*p* < 0.001) were observed in the ∑MUFA with respect to the type of muscle; the breast muscles showed higher contents of this type of fatty acids. Specifically, the BBK treatment was different (*p* ≤ 0.05) from the other treatments.

The profile of the PUFAs in this study is shown in Table 3.

In decreasing order of percentage, the main PUFAs in raw and heat-treated muscles were: C18:2 *n*-6, C 20:4 *n*-6, C 18:3 *n*-3, C18:2 *n*-9, and C 18:3 *n*-6, and they responded differently to heat treatments. Both muscle type (*p* = 0.15–0.76, depending on fatty acid type) and muscle type and heat treatment interaction (*p* = 0.276–0.720) did not affect (*p* > 0.001) the PUFAs profile although the application of heat treatments did (*p* ≤ 0.001).

Linoleic acid (C:18:2 *n*-6) decreased (*p* ≤ 0.001) upon application of the heat treatment, and the raw samples had a higher content of this PUFA (BRM and LRM, 23.07% and 24.46%, respectively), followed by the boiled samples (BBL: 21.17% and LBL: 21.57%) and, lastly, the baked samples (BBL: 19.05% and LBK: 19.65%). Linoleic fatty acid (C:18:2 *n*-9), it was different (*p* ≤ 0.05); the LBK treatment (0.25%) was observed to have a higher content compared to the other treatments. The highest losses of linolenic acid (C:18:3 *n*-3) were with the baked method (BBK and LBK: 0.33%), which was different (*p* ≤ 0.001) from the muscles in raw meat and the boiled treatment. The heat treatment affected (*p* ≤ 0.05) the performance of arachidonic acid (C:20:4 *n*-6); it is the second PUFA with the highest content after linoleic acid. The *n*-6 series of PUFAs found in this study were in a higher percentage in raw meat (BRM = 27.86 and LRM = 29.14), and either heat treatment (boiled or baked) applied negatively affected (*p* ≤ 0.001) the content of ∑PUFA *n*-6. A similar situation was found in the total PUFAs since the *n*-6 series represented 98% of ∑PUFA, being lower (*p* ≤ 0.001) for the BBK (22.58%) and LBK (23.79) treatments and, to a lesser extent, the BBL (25.66%) and LBL (25.06%) treatments in comparison to raw meat (BRM = 28.19, LRM = 29.5).

The results of the analysis related to the lipid indices for health are shown in Table 4.

In general, no differences (*p* = 0.224) were found for NVI, but differences (*p* < 0.001) were observed in the rest of the indices (∑UFA, ∑DFA, ∑OFA, ∑EFA, ∑DFA/∑OFA, ∑UFA/∑SFA, ∑PUFA/∑SFA, and AI). The raw muscle samples (BRM and LRM) had a higher content of fatty acids that make up these indices, followed by those that were cooked with a boiled treatment (BBL and LBL) and, finally, those baked (BBK and LBK).

The first index of lipids for health with the highest content is DFA; the BRM treatment (69.24%) was different (*p* ≤ 0.05) from the LBL (65.06%) and similar (*p* > 0.05) to the rest of the treatments. The second index with the highest content is the UFA in the same way as the previous one, and the BRM treatment (53.69%) was different (*p* ≤ 0.05) from the LBL (48.68%) and LBK (49.65%) treatments and similar (*p* > 0.05) with the other treatments. Unlike these indices, the LRM treatment (29.14%) had a higher EFA concentration than the rest of the treatments and was different (*p* ≤ 0.05) from the thermal baked treatments (BBK = 22.14 and LBK = 23.24). The LRM was similar (*p* > 0.05) with respect to the ratio of ∑DFA/∑OFA to the BRM, BBL, and LBL but significantly different (*p* ≤ 0.05) from the BBK and LBK treatments. One of the most important ratios is ∑UFA/∑SFA; in this study, a higher ratio was found in raw treatments (BRM = 3.13 and LRM = 3.35); however, the heat treatments caused a significant decrease in the proportion of these fatty acids. The application of heat treatment (cooked and baked) in both types of muscles affected negatively (*p* ≤ 0.001) the ratio ∑PUFA/∑SFA with respect to raw meat; however, there were no differences according to muscle type and its interaction with treatment (*p* > 0.05). Although no differences were found (*p* = 0.224) in the nutritive index (NVI), the LBL treatment maintained the highest value (12.83%), and the lowest value was that of BBK (9.8%). In all the nutritional quality indices described, a decrease in the index of raw muscles was observed, as they were exposed to the thermal treatment of boiling and baking, and unlike the proportion ∑OFA, AI index and TI index increased as the heat treatments were applied. The samples that make up the ∑OFA presented lower contents in raw muscles (BRM = 22.13% and LRM = 22.09%), similar to boiled muscles (BBL = 22.68%, LBL = 25.06%) and higher in baked muscles (BBK = 24.29%, LBK = 23.36%). A higher concentration in the atherogenic index (AI) was observed in the muscles that received baked heat treatment, which was significantly different (*p* ≤ 0.001) from the cooking treatments and raw muscles. In contrast, the thrombogenic index maintained a significantly higher concentration (*p* < 0.001) in both cooking treatments with respect to raw muscles, with no statistical differences in the type of muscle (*p* > 0.926) and the interaction of treatment and type of muscle (*p* > 0.941).

## 4. Discussion

The consumption of foods high in saturated fats and cholesterol of animal origin can cause coronary diseases [17]; which generates interest in the composition of fatty acids to develop ways to produce healthier meat with a higher ratio of polyunsaturated fatty acids (PUFA) to saturated fatty acids (SFA) and a more favorable balance between PUFA *n*-6 and *n*-3 [18]. The values of *n*-6/*n*-3 ratio found in the present study were higher than those obtained by Gálvez et al. [19] in commercial turkey. These proportions are used to judge the nutritional value of meat and the fat health index for human consumption [9]. However, a key factor in the use of fatty acids is the preparation method before consumption of meat, the cooking methods used, and the conditions of heating speed. The differences observed in this study regarding the fatty acid content could be associated with cooking time and temperature. Previous studies identified that these factors affect the chemical composition and nutritional value, which vary depending on different factors, such as the animal species, type of cut or meat products, and heat treatment techniques [16,20,21,22]. In addition, Werénska et al. [9] mentioned that oxidation, hydrolysis, and polymerization are some of the chemical reactions that lipids can undergo, with polyunsaturated lipids being more susceptible to oxidation and unsaturated lipids being unstable to heat as the degree of saturation increases [23]. King salmon, one of the species with a high concentration of unsaturated fatty acids, was evaluated through different thermal treatments and common preparation techniques (raw, poached, steamed, microwaved, fried, baked, and fried with oil) before its consumption [22] to achieve the optimal preparation with the best sensory quality. There were differences between the methods, with an increase in PUFA. The only difference was the oil-fried treatment due to its absorption of linolenic acid from the frying. Danowska-Oziewicz et al. [23], in a study on turkeys, reported that the higher the air saturation through the application of the cooking treatment, the higher the concentration of saturated acids (34.45–40.51%) and polyunsaturated acids (27.87–28.35%); however, monounsaturated acids decreased (3.67–32.45%).

Lipid oxidation depends on the nature of the triglycerides, antioxidant, and metal ion composition. The differences observed in the performance of fatty acids, specifically PUFAs, show that increasing the heat and cooking time of any type of muscle produces an oxidative degradation of fatty acids; therefore, temperature and time are important factors that lead to controlled oxidation of lipids [24]. Kamal et al. [25] found that, in lamb meat, the SFA and MUFA rates decreased, while that of PUFA increased after cooking. The decrease in SFA mainly concerns C16:0 and C18:0, whereas that of MUFA is mainly related to a decrease in C18:1. These two decreases are due to a loss of the main molecular species of triglycerides (OOP, SOP, POP, OOS) consisting mainly of these fatty acids.

NVI values are lower than those recommended; this may be due to the high proportions of the acids that make up this index, as is the case of stearic acid (C:18:0) and palmitoleic acid (C:16:1). The atherogenic index (AI) indicate a potential for stimulating platelet aggregation. Thus, the smaller the AI value, the greater the protective potential for coronary artery disease. In human health, the AI, which is less than 1.0, in the diet, is recommended [9]. In this study, the AI value did not show differences due to the effect of heat treatments and ranged from 0.44 to 0.55. These results are consistent with those reported by Gálvez et al. [19] in the breast (AI = 0.43) and thigh (AI = 0.46) muscles of commercial male turkeys. The thrombogenic index (TI) determines the balanced fatty acid content and measures the thrombotic capacity of a food. Human dietary recommendations for the thrombogenic index are similar to those for AI. The TI values in this study were higher in the cooking and baking treatments, and differences were found with respect to raw muscles. These TI values are lower than those reported by Krawczyk et al. [26] in a study on turkeys fed with different percentages of yellow lupine seed, finding ranges of 0.63 to 0.76 TI values. It is well-known that myristic and palmitic acids are among the most atherogenic agents, while stearic acid is believed to be neutral regarding atherogenicity, which is instead considered thrombogenic [11]. A ΣPUFA/ΣSFA ratio greater than 0.45 is recommended in the human diet to prevent the development of cardiovascular diseases and some other diseases, including cancer. Foods with ΣPUFA/ΣSFA ratios below 0.45 have been considered undesirable for the human diet due to their potential to induce an increase in blood cholesterol [27]; the ratio found in this study was higher than recommended. Lipid oxidation has negative effects on meat quality of broilers [28]. However, an increase in the amount of *n*-3 PUFA in food, especially docosahexaenoic acid (DHA) and eicosapentaenoic acid (EPA), can confer greater susceptibility to lipid oxidation, and oxidative deterioration negatively affects the sensory quality of the products, including odors or flavors during storage [29].

No omega-3 fatty acids other than C18:3n3 (ALA) were detected. This could be due to a genetic difference between commercial turkeys and native Mexican guajolotes, a difference in the type of feed, or a combination of both factors. In this regard, the fatty acid profile of turkeys found in the literature is often from commercial animals with a strong directional genetic selection that seeks to improve productive aspects, such as rapid growth and meat quality. In contrast, native Mexican guajolotes, such as those in this study, are developed without any structured selection program. Likewise, in the traditional production system, guajolotes are not under the direct reproductive control of humans; they are raised in a completely free-ranging system. Recently, it has been shown that there is a differentiation in genome evolution between the commercial turkey and the native Mexican guajolote. Commercial turkeys have improved their productive aptitude, particularly in meat production [8]. This is due to the controlled breeding conditions of this type of turkey. Meanwhile, the native Mexican guajolote has a better ability to adapt to the natural environment. In addition, the native Mexican guajolote has a greater genetic variability compared to commercial turkey breeds as a consequence of the long period of adaptation to the adverse environmental conditions that characterize Mexico [7].

On the other hand, the native Mexican guajolote’s feed is based on local cereals (corn, sorghum, wheat, among others) and organic ingredients collected during grazing (seeds, grasses, herbs, fruits, vegetables, and edible insects). In contrast, commercial turkeys are fed totally mixed rations, which may include additives, some of which are included to change the fatty acid profile of the meat. This situation causes a variation in the concentration of fatty acids in commercial turkey meat. For example, the concentration of C20:5 n3 (EPA) in breast meat varied from 0.05 g/100 g AG [30] to 0.15 g/100 g AG [31] and 0.20 g/100 g AG [19] to not being detected by Baggio et al. [32]. In the case of C22:6 n3 (DHA), the concentration ranged from 0.25 g/100 g AG [32] to 0.15 g/100 g AG [30] and 0.93 g/100 g AG [19] to not being detected by Göncü-Karakök et al. [31]. Similarly, studies of meat nutritional quality and fatty acid profile have focused on commercial turkeys to the best of our knowledge; this research would be the first report of meat quality and fatty acid profile in native Mexican guajolote.

## 5. Conclusions

The content of MUFAs in native guajolote breast shows a higher proportion of fatty acids than in the leg. Heat treatments applied to the breast or leg increase the content of SFA and MUFAs in raw meat. Baking is less favorable for both types of muscle. Boiling or baking the breast or leg of native guajolote deteriorates PUFAs but increases the OFA and AI indices.

## Figures and Tables

**Table 1:** Saturated fatty acid (SFA) profile of raw and cooked native guajolote meat (% of total fatty acids).

Fatty Acid	Raw Meat		Heat Treatment				*p*-Value (*p* < 0.05)		
			Boiled		Baked		M	MCK	MxCK
	Breast (BRM)	Leg (LRM)	Breast (BBL)	Leg (LBL)	Breast (BBK)	Leg (LBK)			
C 12:0	0.15 ± 0.11 ^b^	0.11 ± 0.03 ^b^	0.19 ± 0.06 ^b^	0.14 ± 0.07 ^b^	0.34 ± 0.15 ^a^	0.25 ± 0.13 ^a,b^	0.048	<0.001	0.784
C 14:0	0.64 ± 0.08	0.64 ± 0.15	0.60 ± 0.13	0.60 ± 0.09	0.61 ± 0.10	0.59 ± 0.20	0.614	0.756	0.961
C 15:0	3.69 ± 1.31	3.08 ± 0.86	4.22 ± 1.90	3.65 ± 0.94	3.54 ± 1.80	3.71 ± 1.66	0.384	0.501	0.663
C 16:0	21.48 ± 0.97 ^b,c^	20.38 ± 1.06 ^c^	22.07 ± 1.56 ^a,b,c^	21.54 ± 1.48 ^b,c^	23.68 ± 2.17 ^a^	22.77 ± 1.56 ^a,b^	0.037	<0.001	0.830
C 17:0	1.19 ± 0.42	0.79 ± 0.20	1.05 ± 0.55	1.00 ± 0.20	0.88 ± 0.62	1.05 ± 0.61	0.447	0.924	0.184
C 18:0	9.49 ± 1.26 ^b^	12.39 ± 1.50 ^a,b^	13.10 ± 2.34 ^a^	13.50 ± 2.03 ^a^	13.05 ± 3.16 ^a^	13.87 ± 2.33 ^a^	0.018	<0.001	0.160
C 20:0	0.10 ± 0.03	0.11 ± 0.04	0.10 ± 0.05	0.13 ± 0.05	0.10 ± 0.06	0.11 ± 0.02	0.166	0.649	0.903
∑ SFA	36.74 ± 2.14 ^c^	37.50 ± 2.39 ^b,c^	41.33 ± 3.33 ^a,b^	40.52 ± 2.70 ^a,b,c^	42.20 ± 3.54 ^a^	42.35 ± 3.51 ^a^	0.962	<0.001	0.712

^a–c^ Different letters in rows means statistically significant differences between group average, including thermal treatment (*p* ≤ 0.05). M, meat differences (breast or leg); MCK, differences between raw meat and heat treatment; MxCK, interaction between meat and raw meat and heat treatment; ∑ SFA, sum of saturated fatty acids.

**Table 2:** Monounsaturated fatty acid (MUFA) profile of raw and cooked native guajolote meat (% of total fatty acids).

Fatty Acid	Raw Meat (RM)		Heat Treatment				*p*-Value (*p* < 0.05)		
			Boiled (BL)		Baked (BK)		M	MCK	MxCK
	Breast (BRM)	Leg (LRM)	Breast (BBL)	Leg (LBL)	Breast (BBK)	Leg (LBK)			
C 14:1	0.12 ± 0.04 ^a,b^	0.09 ± 0.01 ^b^	0.12 ± 0.04 ^a,b^	0.11 ± 0.05 ^a,b^	0.15 ± 0.03 ^a^	0.15 ± 0.05 ^a,b^	0.257	<0.001	0.781
C 16:1	3.51 ± 1.27 ^b,c^	3.19 ± 0.69 ^b,c^	3.71 ± 1.13 ^b,c^	2.96 ± 0.50 ^b^	4.37 ± 1.40 ^a^	3.22 ± 0.74 ^b,c^	<0.001	0.285	0.453
C 17:1	0.36 ± 0.10 ^b^	0.29 ± 0.06 ^b^	0.42 ± 0.24 ^b^	0.35 ± 0.06 ^b^	0.89 ± 0.65 ^a^	0.71 ± 0.51 ^a,b^	0.256	<0.001	0.843
C 18:1 *Cis*	26.51 ± 2.54 ^a^	23.86 ± 2.37 ^a,b^	25.16 ± 3.68 ^a,b^	23.56 ± 2.70 ^a,b^	25.90 ± 2.42 ^a,b^	22.43 ± 1.33 ^b^	0.446	<0.001	0.550
C 18:1 *Trans*	0.19 ± 0.07 ^b^	0.29 ± 0.07 ^a^	0.17 ± 0.07 ^b^	0.17 ± 0.05 ^b^	0.20 ± 0.06 ^a,b^	0.23 ± 0.07 ^a,b^	0.026	0.009	0.099
C 20:1	0.19 ± 0.06	0.21 ± 0.08	0.21 ± 0.08	0.22 ± 0.03	0.20 ± 0.04	0.24 ± 0.04	0.104	0.680	0.787
C 24:1	0.64 ± 0.24 ^a^	0.42 ± 0.17 ^a,b^	0.43 ± 0.16 ^a,b^	0.41 ± 0.06 ^b^	0.36 ± 0.16 ^b^	0.37 ± 0.10 ^b^	0.090	<0.001	0.064
∑MUFA	31.55 ± 3.19 ^a,b^	28.38 ± 2.46 ^a,b,c^	30.25 ± 3.81 ^a,b,c^	27.82 ± 2.92 ^b,c^	32.11 ± 2.97 ^a^	27.39 ± 1.47 ^c^	<0.001	0.574	0.475

^a–c^ Different letters in rows means statistically significant differences between group average, including thermal treatment (*p* ≤ 0.05). M, meat differences (breast or leg); MCK, differences between raw meat and heat treatment; MxCK, interaction between meat and raw meat and heat treatment; ∑MUFA, sum of monounsaturated fatty acids.

**Table 3:** Polyunsaturated fatty acid (PUFA) profile of raw and cooked native guajolote meat (% of total fatty acids).

Fatty Acid	Raw Meat (RM)		Heat Treatment				*p*-Value (*p* < 0.05)		
			Boiled (BL)		Baked (BK)		M	MCK	MxCK
	Breast (BRM)	Leg (LRM)	Breast (BBL)	Leg (LBL)	Breast (BBK)	Leg (LBK)			
C 18:2 *n-6*	23.07 ± 2.25 ^a,b^	24.46 ± 1.56 ^a^	21.17 ± 2.59 ^b,c^	21.57 ± 2.16 ^b,c^	19.05 ± 1.44 ^c^	19.65 ± 2.06 ^c^	0.148	<0.001	0.720
C 18:2 *n-9*	0.11 ± 0.05 ^a^	0.16 ± 0.03 ^a,b^	0.19 ± 0.13 ^a,b^	0.20 ± 0.11 ^a,b^	0.17 ± 0.07 ^a,b^	0.25 ± 0.10 ^a^	0.052	0.031	0.485
C 18:3 *n-3*	0.58 ± 0.16 ^a,b^	0.70 ± 0.19 ^a^	0.48 ± 0.23 ^a,b^	0.58 ± 0.23 ^a,b^	0.33 ± 0.11 ^b^	0.33 ± 0.15 ^b^	0.150	<0.001	0.548
C 18:3 *n-6*	0.20 ± 0.09 ^a^	0.19 ± 0.08 ^a^	0.29 ± 0.15 ^a,b^	0.42 ± 0.16 ^a^	0.27 ± 0.15 ^a,b^	0.29 ± 0.17 ^a,b^	0.230	<0.001	0.276
C 20:4 *n-6*	4.20 ± 1.39	3.97 ± 1.20	3.51 ± 1.53	2.90 ± 1.20	2.75 ± 1.59	3.25 ± 1.55	0.761	0.048	0.475
∑PUFA *n-6*	27.86 ± 1.87 ^a,b^	29.14 ± 1.89 ^a^	25.17 ± 3.33 ^b,c^	25.06 ± 2.47 ^b,c^	22.14 ± 2.14 ^c^	23.24 ± 2.14 ^c^	0.229	<0.001	0.603
∑PUFA	28.19 ± 1.87 ^a,b^	29.50 ± 1.89 ^a^	25.66 ± 3.42 ^b,c^	25.69 ± 2.58 ^b,c^	22.58 ± 2.14 ^c^	23.79 ± 1.55 ^c^	0.173	<0.001	0.635

^a–c^ Different letters in rows means statistically significant differences between group average, including thermal treatment (*p* ≤ 0.05). M, meat differences (breast or leg); MCK, differences between raw meat and heat treatment; MxCK, interaction between meat and raw meat and heat treatment; ∑PUFA, sum of polyunsaturated fatty acids.

**Table 4:** Nutritional quality indices of the lipids in raw and cooked native guajolote meat (% of total fatty acids).

Fatty Acid	Raw Meat (RM)		Heat Treatment				*p*-Value (*p* < 0.05)		
			Boiled (BL)		Baked (BK)		M	MCK	MxCK
	Breast (BRM)	Leg (LRM)	Breast (BBL)	Leg (LBL)	Breast (BBK)	Leg (LBK)			
∑UFA	53.69 ± 4.25 ^a^	52.32 ± 2.78 ^a,b^	50.54 ± 4.16 ^a,b^	48.68 ± 2.68 ^b,c^	49.67 ± 3.70 ^a,b,c^	45.65 ± 3.02 ^c^	0.014	<0.001	0.489
∑DFA	69.24 ± 2.03 ^a^	70.29 ± 2.58 ^a^	69.02 ± 3.33 ^a^	67.02 ± 3.87 ^a,b^	67.71 ± 2.50 ^a,b^	65.06 ± 2.67 ^c^	0.116	<0.001	0.108
∑OFA	22.13 ± 1.02 ^b,c^	21.09 ± 1.12 ^c^	22.68 ±1.63 ^a,b,c^	22.15 ± 1.49 ^b,c^	24.29 ± 2.20 ^a^	23.36 ± 1.71 ^a,b^	0.042	<0.001	0.840
∑EFA	27.86 ± 1.87 ^a,b^	29.14 ± 1.89 ^a^	25.17 ± 3.33 ^b,c^	25.06 ± 2.74 ^b,c^	22.14 ± 2.14 ^c^	23.24 ± 1.66 ^c^	0.229	<0.001	0.603
∑DFA/∑OFA	3.13 ± 0.18 ^a,b^	3.35 ± 0.23 ^a^	3.06 ± 0.35 ^a,b^	3.04 ± 0.31 ^a,b^	2.81 ± 0.36 ^b^	2.79 ± 0.23 ^b^	0.441	<0.001	0.341
∑UFA/∑SFA	1.40 ± 0.20 ^a^	1.39 ± 0.13 ^a,b^	1.23 ± 0.20 ^b,c^	1.20 ± 0.11 ^b,c^	1.19 ± 0.18 ^b,c^	1.08 ± 0.15 ^c^	0.131	<0.001	0.800
∑PUFA/∑SFA	0.76 ± 0.72 ^a^	0.78 ± 0.07 ^a^	0.62 ± 0.09 ^b^	0.63 ± 0.08 ^b^	0.53 ± 0.07 ^b^	0.56 ± 0.08 ^b^	0.356	<0.001	0.960
NVI	11.46 ± 3.97	11.76 ± 2.51	11.58 ± 5.68	12.83 ± 2.49	9.80 ± 3.34	11.80 ± 2.85	0.502	0.224	0.776
AI	0.45 ± 0.02	0.44 ± 0.02	0.49 ± 0.04	0.49 ± 04	0.53 ± 0.06	0.55 ± 0.04	0.631	<0.001	0.539
TI	0.31 ± 0.07 ^b^	0.32 ± 0.08 ^b^	0.39 ± 0.01 ^a^	0.39 ± 0.01 ^a^	0.44 ± 0.01 ^a^	0.44 ± 0.01 ^a^	0.926	<0.001	0.941

^a–c^ Different letters in rows means statistically significant differences between group average, including thermal treatment (*p* ≤ 0.05). M, meat differences (breast or leg); MCK, differences between raw meat and heat treatment; MxCK, interaction between meat and raw meat and heat treatment; ∑UFA, sum of unsaturated fatty acids; ∑DFA, dietary fatty acids; ∑OFA, hypercholesterolemic fatty acids; ∑EFA, essential fatty acids; NVI, nutritive value index; AI, atherogenic index; TI, thrombogenic index.

## Data Availability

The data presented in this study are available on request from the corresponding author.
